# A Comprehensive Analysis Revealing BUB1B as a Potential Prognostic and Immunological Biomarker in Lung Adenocarcinoma

**DOI:** 10.3390/ijms26052061

**Published:** 2025-02-26

**Authors:** Zhenzhen Hao, Fei An, Wanting Zhang, Xiaoshuang Zhu, Shihao Meng, Bo Zhao

**Affiliations:** Institute of Biochemistry and Molecular Biology, College of Life and Health Sciences, Northeastern University, Shenyang 110819, China; 1710065@stu.neu.edu.cn (Z.H.); 2201435@stu.neu.edu.cn (F.A.); 2110482@stu.neu.edu.cn (W.Z.); 2010494@stu.neu.edu.cn (X.Z.); 2310514@stu.neu.edu.cn (S.M.)

**Keywords:** pan-cancer, BUB1B, immune microenvironment, prognosis, LUAD, biomarker

## Abstract

BUB1B, a member of the spindle assembly checkpoint family known as BUB1 mitotic checkpoint serine/threonine kinase B, has been associated with the promotion of tumor progression. Nevertheless, its specific contributions to tumorigenesis remain largely unexplored. This study seeks to offer a systematic and comprehensive analysis of the role of BUB1B in the progression of various cancers, with a particular focus on lung adenocarcinoma, utilizing a range of databases. We investigated BUB1B’s role in pan-cancer using TCGA data, analyzing it with platforms like HPA, TIMER, TISIDB, GEPIA, cBioPortal, GDC, LinkedOmics, and CancerSEA. Additionally, we assessed BUB1B’s impact on lung adenocarcinoma proliferation and migration through CCK-8, wound healing, transwell assays and Western blot analysis. This study found that BUB1B was upregulated in most cancers and was significantly linked to patient prognosis. Its expression correlated with immune cell infiltration and genetic markers of immunomodulators across different cancers. BUB1B was involved in the acute inflammatory response and IgA production pathways but negatively correlated with inflammation in lung adenocarcinoma. Moreover, the siRNA-mediated knockdown of BUB1B resulted in the inhibition of proliferation and migration of lung cancer cells in vitro. This study underscores the potential of BUB1B as a biomarker and a promising therapeutic target for patients with lung adenocarcinoma.

## 1. Introduction

In contemporary times, cancer poses a significant threat to public health. Despite substantial advancements in cancer treatment modalities such as surgery, chemotherapy, and radiotherapy, the 5-year overall survival (OS) rate for patients remains suboptimal. In recent years, immunotherapy has achieved considerable success in cancer treatment, exemplified by the clinical efficacy of immune checkpoint inhibitors [1,2,3,4]. Furthermore, the rapid advancement of the TCGA database has facilitated more comprehensive analyses of the correlation and impact of individual genes on cancer prognosis and immune infiltration. Consequently, there is a pressing need to identify novel diagnostic and prognostic biomarkers for cancer. Additionally, the significant success of immunotherapy has underscored the importance of immune-related biomarkers.

Cancer immunotherapy, which utilizes the immune system to identify and eradicate cancer, has become a vital part of cancer treatment. Furthermore, the tumor microenvironment (TME) consists of diverse cellular components and an extracellular matrix, with infiltrating immune cells constituting a significant portion [5,6,7]. The interaction between tumor cells and immune cells is acknowledged as a critical determinant of tumor escape, progression, and response to therapy [8,9]. The pre-existing antitumor adaptive immune contexture encompasses various elements, including antigen processing, immune checkpoints/immunomodulators, effector cells, and suppressor cells. A comprehensive understanding of the tumor microenvironment (TME) could facilitate deeper insights into the mechanisms underlying tumor development and enhance therapeutic efficacy.

Lung adenocarcinoma (LUAD), a common lung cancer subtype, has advanced in immunotherapy, notably with immune checkpoint inhibitors (ICIs) that target PD-1 and PD-L1 to boost the immune response and improve survival [10]. However, not all patients benefit, as the effectiveness of immunotherapy is linked to immune cell infiltration patterns, particularly CD8+ T cells, in the tumor microenvironment. Cytokines and chemokines regulate this infiltration [11]. In LUAD immunotherapy, combination strategies like radiotherapy and immunotherapy are gaining attention for boosting immune responses, though they may cause immune escape. Identifying molecular markers to predict combination therapy responses is essential [12]. Certain long non-coding RNAs (lncRNAs) are linked to immune infiltration and prognosis, offering new diagnostic and treatment insights [13]. Overall, LUAD immunotherapy is evolving with immune checkpoint inhibitors, immune cell infiltration regulation, and combination strategies. Future research aims to uncover mechanisms, discover new biomarkers, and improve treatment effectiveness and patient quality of life.

BUB1B (BUB1 mitotic checkpoint serine/threonine kinase B) is a member of the spindle family, which plays a crucial role in the attachment of chromosomes to mitotic spindles during the process of mitosis [14]. This multifunctional protein forms complexes with CDC20, BUB3, and MAD2 to inhibit the activity of the anaphase-promoting complex or cyclosome [15]. Given the essential function of BUB1b in the regulation of chromosome segregation, abnormal expression levels of BUB1b may result in chromosomal instability and an increased incidence of cancer [16]. Research has demonstrated that the overexpression of BUB1B is associated with unfavorable prognoses in various cancer patients, indicating its potential significance in tumor metastasis and treatment resistance. Consequently, BUB1B may serve as a valuable molecular marker. For instance, BUB1B overexpression has been shown to enhance proliferation and invasion in endometrial and gastric cancers [17,18]. Elevated levels of BUB1B facilitate the proliferation of multiple myeloma cells through the CDC20/CCNB signaling axis [19,20]. Additionally, BUB1B contributes to the progression of hepatocellular carcinoma by activating the mTORC1 signaling pathway [21]. Moreover, elevated BUB1B expression has been associated with poorer survival outcomes in patients with renal cell carcinoma, prostate cancer, thyroid carcinoma, and pancreatic ductal adenocarcinoma [22,23,24,25].

In this study, we conducted a comprehensive analysis to assess the potential utility of BUB1B in relation to cancer pathological staging and prognosis. Furthermore, we performed an enrichment analysis of genes co-expressed with BUB1B. We also investigated the correlation between BUB1B expression levels and the tumor microenvironment (TME), including the expression of immunomodulators and the infiltration of immune cells. These efforts aim to enhance the understanding of the significance and potential role of BUB1B across various tumor types.

## 2. Results

### 2.1. BUB1B Was Upregulated in Multiple Human Cancers

**Figure 1:**
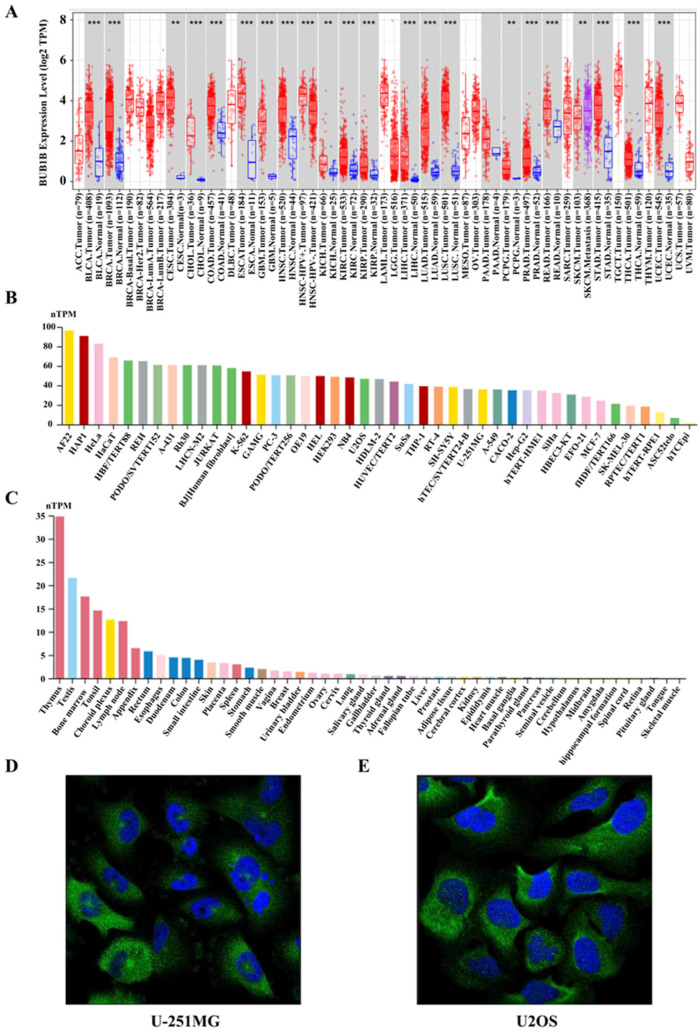
Pan-cancer analysis of BUB1B expression. (**A**) Differential expression of BUB1B between tumor and normal tissues in pan-cancer analysis. (**B**,**C**) Expression of BUB1B in various cancer cell lines and tissues. (**D,E**) Cellular localization of BUB1B from U-251MG and U2OS. ** *p* < 0.01, *** *p* < 0.001.

Initially, to investigate the expression level of BUB1B, we conducted an analysis using the TIMER 2.0 database on the TCGA datasets. The findings indicated that BUB1B was upregulated in the majority of cancers, including BLCA, BRCA, CESC, CHOL, COAD, ESCA, GBM, HNSC, KICH, KIRC, KIRP, LIHC, LUAD, LUSC, PCPG, PRAD, READ, SKCM, STAD, THCA, and UCEC (*p* < 0.05) (Figure 1A). Furthermore, it was observed that BUB1B was highly expressed in most cancer cell lines (Figure 1B). Histopathological analyses revealed that BUB1B was predominantly expressed in the thymus (Figure 1C), with a primary localization to the cytosol and basal body (Figure 1D). Next, we explored the correlation between BUB1B expression and pathological stage in the GEPIA2 database. BUB1B expression was markedly related to the stage of 11 types of cancer, including LUAD (Figure S1G). The findings demonstrate a significant upregulation of BUB1B expression across multiple cancer types, suggesting its potential as a crucial biomarker for cancer diagnosis and therapeutic targeting.

### 2.2. Pan-Cancer Analysis of the Diagnostic and Prognostic Value of BUB1B

**Figure 2:**
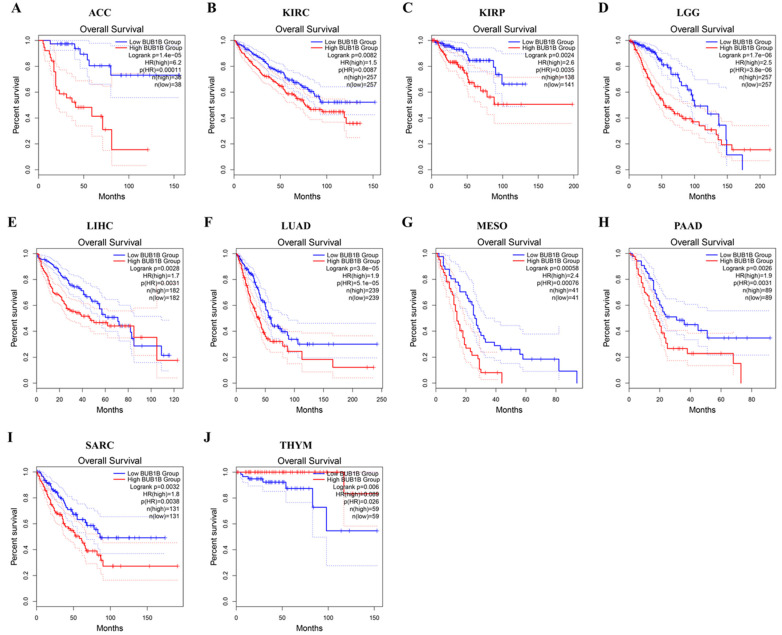
BUB1B expression correlates with overall survival time (OS). GEPIA2 analyses of the association between BUB1B expression and OS in (**A**) ACC, (**B**) KIRC, (**C**) KIRP, (**D**) LGG, (**E**) LIHC, (**F**) LUAD, (**G**) MESO, (**H**) PAAD, (**I**) SARC, and (**J**) THYM.

Subsequently, we analyzed the diagnostic value of BUB1B in various cancers using the ROC curve. As shown in Figure S2, BUB1B may act as a perfect diagnostic marker, especially in LUAD (AUC = 0.977). To further explore the relationship between BUB1B expression and cancer prognosis, we conducted a survival analysis across 33 cancer types utilizing overall survival (OS) as the metric. GEPIA2 analysis revealed that elevated BUB1B expression was associated with poorer OS in ACC, KIRC, KIRP, LGG, LIHC, LUAD, MESO, PAAD, and SARC (Figure 2A–I, *p* < 0.05). Conversely, increased BUB1B expression correlated with improved OS in THYM (*p* < 0.05) (Figure 2J). These findings suggest that BUB1B expression was significantly associated with tumor prognosis.

### 2.3. The Characteristics of BUB1B Mutations in the TCGA Pan-Cancer Cohort

**Figure 3:**
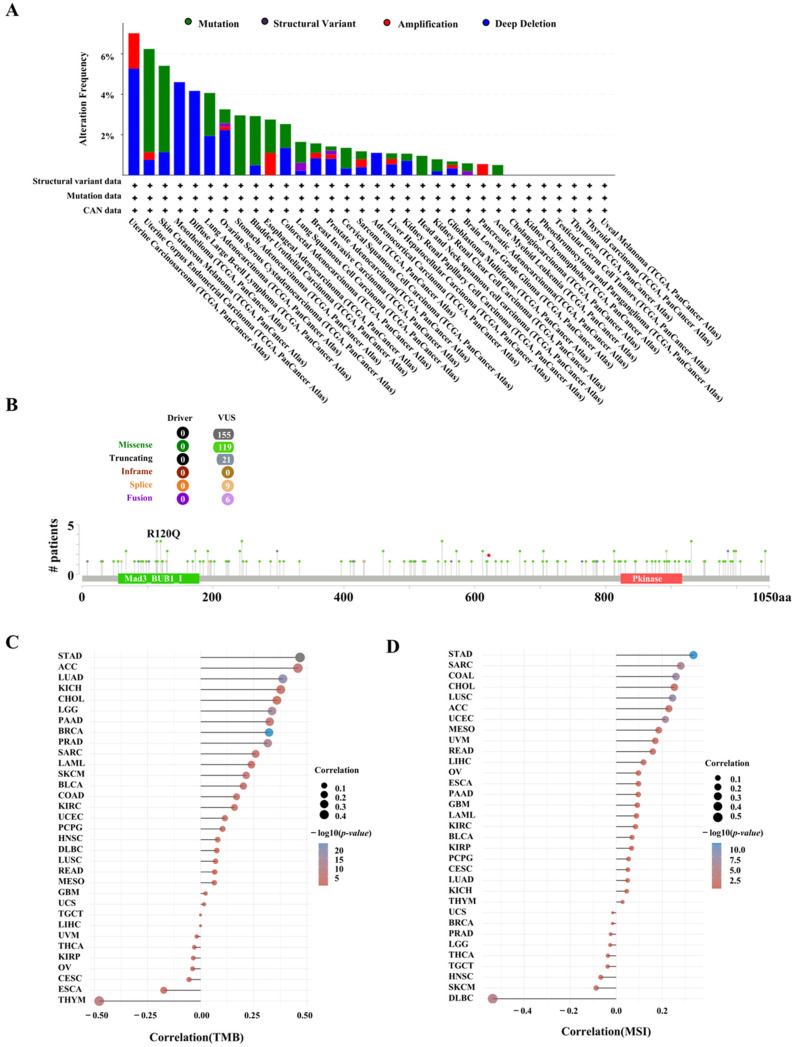
Correlation between BUB1B expression and mutations in various cancer types. (**A**) Landscape of BUB1B mutation in 32 cancer types, (**B**) the subtypes and distributions of BUB1B somatic mutations, (**C**,**D**) Spearman correlation analysis for TMB, MSI, and BUB1B gene expression. In the figure, the horizontal axis represents the correlation coefficient between the genes and TMB, and the vertical axis represents the different tumors. The size of the dots in the figure represents the correlation coefficient, and the different colors represent the significance of the *p* value. The bluer the color in the diagram, the smaller the *p* value.

Subsequently, we examined the alteration status of BUB1B across the TCGA cohorts. As illustrated in Figure 3A, BUB1B alterations were identified in 32 cancer types, with uterine carcinosarcoma exhibiting the highest frequency at 7.02%. Other notable frequencies were observed in uterine corpus endometrial carcinoma (6.24%), skin cutaneous melanoma (5.41%), mesothelioma (4.6%), diffuse large B-cell lymphoma (4.17%), and lung adenocarcinoma (4.06%). The alteration sites, types, and quantities of BUB1B are further illustrated in Figure 3B. Additionally, the expression of BUB1B demonstrates a correlation with tumor mutational burden (TMB) across nine cancer types, as depicted in Figure 3C. Furthermore, we found that BUB1B expression was positively correlated with MSI in five cancers but negatively correlated in DLBC (Figure 3D).

### 2.4. BUB1B Is Related to Immune Microenvironment in Pan-Cancer

**Figure 4:**
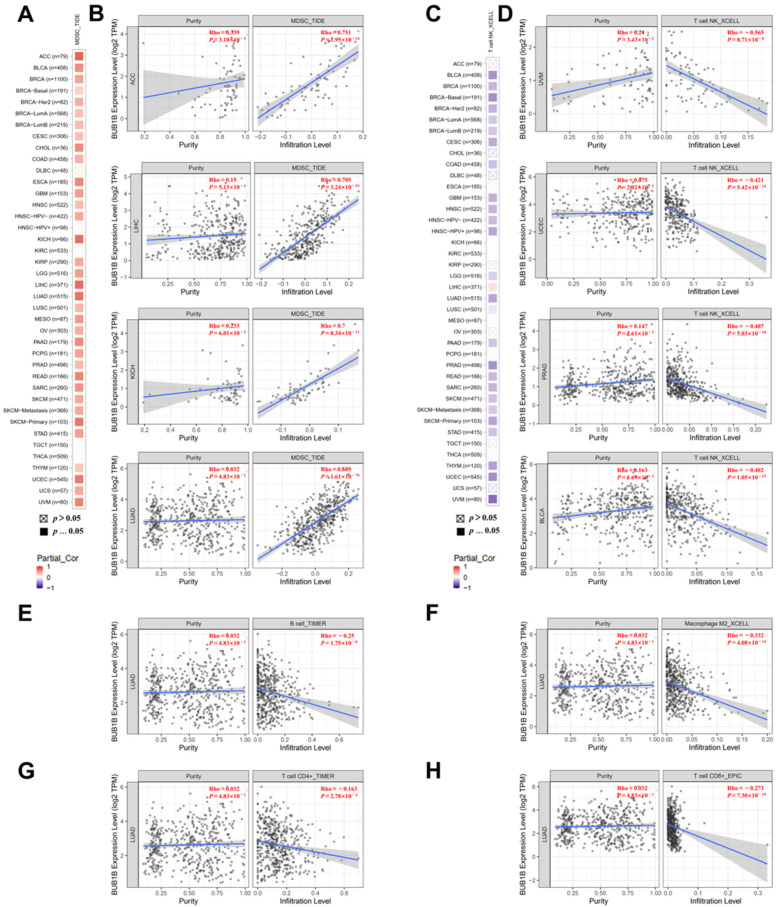
The association between BUB1B expression and immune cell infiltration. (**A**,**B**) BUB1B expression is positively associated with MDSC infiltration in pan-cancer. (**C**,**D**) BUB1B expression is positively negative with NKT cell infiltration in pan-cancer. (**E**–**H**) BUB1B expression is negative associated with the infiltration level of B cells, macrophage cells, CD4+ T cells, and CD8+ T cells.

TIMER 2.0 was employed to investigate the potential role of BUB1B in immune cell infiltration. The results indicate a significant positive correlation between the infiltration levels of myeloid-derived suppressor cells (MDSCs) and BUB1B expression in the majority of tumors (Figure 4A). The top four tumors exhibiting this correlation were ACC, LIHC, KICH, and LUAD (*p* < 0.001) (Figure 4B). Conversely, a negative association was observed between T-cell NK infiltration levels and BUB1B expression across most tumors (Figure 4C), with the leading four tumors being UVM, UCEC, PRAD, and BLCA (Figure 4D). Furthermore, we examined the role of BUB1B in immune cell infiltration, specifically in LUAD, revealing a negative association between BUB1B and the infiltration levels of B cells (*p* < 0.001), macrophages (*p* < 0.001), CD4+ T cells (*p* < 0.001), and CD8+ T cells (*p* < 0.001) (Figure 4E–H). Furthermore, an analysis based on the TISIDB database reveals that BUB1B expression in various cancers exhibits significant correlations with immune regulators. It is associated with a diverse array of immune cells, immunomodulators, chemokines, and their receptors (Figure S3). These findings suggest that BUB1B may have a role in modulating the tumor immune microenvironment.

### 2.5. The Function Analysis of BUB1B in LUAD

**Figure 5:**
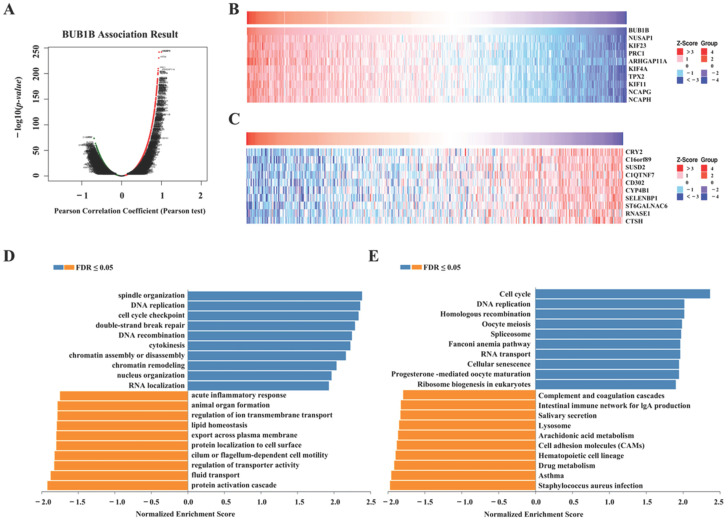
The enrichment analysis of BUB1B co-expression genes in LUAD. (**A**) The BUB1B co-expression genes in LUAD. (**B**,**C**) The top 50 genes positively and negatively correlated to BUB1B. (**D**,**E**) GO and KEGG analysis of BUB1B co-expression genes in the LUAD cohort.

Lung adenocarcinoma represents the most prevalent malignant neoplasm worldwide and poses a significant threat to human health. Our study has demonstrated an association between BUB1B expression and poor prognosis, as well as immune regulation, in lung adenocarcinoma, suggesting a potential role in facilitating tumor immune evasion. Subsequently, we selected lung adenocarcinoma (LUAD) to further investigate the biological function of BUB1B using the LinkedOmics database. Figure 5A illustrates the genes positively and negatively correlated with BUB1B, while Figure 5B,C display the top 10 associated genes. Moreover, GO analysis (biological function) demonstrated that BUB1B mainly joined in spindle organization, DNA replication, cell cycle checkpoint, double-strand break repair, DNA recombination, cytokinesis, acute inflammatory response, etc. (Figure 5D). KEGG analysis illustrates enrichment in the cell cycle, DNA replication, homologous recombination, oocyte meiosis, the spliceosome, the Fanconi anemia pathway, RNA transport, cellular senescence, the intestinal immune network for IgA production, etc. (Figure 5E). The results show that BUB1B was not only involved in the function of cancer, but also involved in inflammation and immunity.

### 2.6. Single-Cell Sequence Analysis of the Correlation Between BUB1B Expression and 14 Cancer Functional States in LUAD

**Figure 6:**
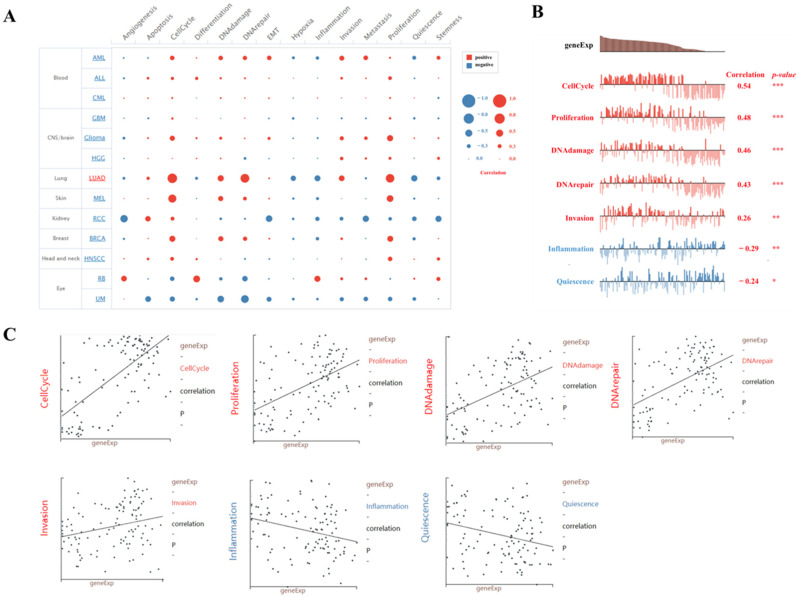
Function of BUB1B in LUAD determined using the CancerSEA database. (**A**) Analysis from the CancerSEA database at single-cell resolution indicated that BUB1B was primarily involved in cell cycle, proliferation, DNA damage, DNA repair, invasion, inflammation, quiescence. (**B**,**C**) Functional relevance in LUAD, BUB1B expression was significantly positively correlated with cell cycle, proliferation, DNA damage, DNA repair, invasion, and was negatively correlated with inflammation, quiescence. The experiments were repeated three times. (* *p* < 0.05, ** *p* < 0.01, *** *p* < 0.001).

Moreover, we conducted an in-depth analysis of the correlation between BUB1B and 14 cancer functional states utilizing single-cell sequencing data from CancerSEA. Our findings indicate that BUB1B exhibits a positive association with cell cycle and proliferation across the majority of tumor types (Figure 6A). Notably, BUB1B demonstrated a negative correlation with the inflammatory status in most tumors, with lung adenocarcinoma (LUAD) being the most prominent example (Figure 6A). In the context of LUAD, BUB1B expression demonstrated a significant positive correlation with cell cycle (correlation coefficient = 0.54), proliferation (correlation coefficient = 0.48), DNA damage (correlation coefficient = 0.46), DNA repair (correlation coefficient = 0.43), and invasion (correlation coefficient = 0.26). Conversely, there was a negative correlation with inflammation (correlation coefficient = −0.29) and quiescence (correlation coefficient = −0.24), as illustrated in Figure 6B,C.

### 2.7. BUB1B Knockdown Suppressed LUAD Cell Proliferation, Migration, and EMT In Vitro

**Figure 7:**
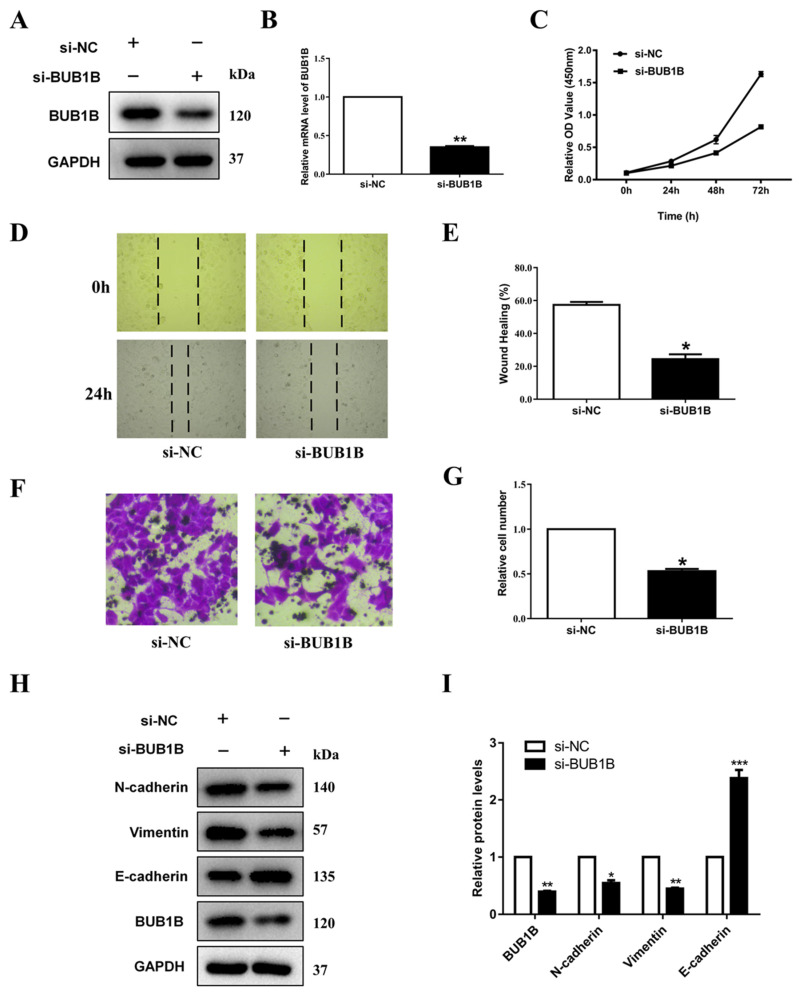
Cellular functions of BUB1B. (**A**,**B**) Western blot and RT-qPCR detection after siRNA-mediated knockdown of BUB1B in A549 cells. (**C**) CCK-8 assay results showing the decrease in the viability of A549 cells upon the knockdown of BUB1B. (**D**,**E**) Wound healing test. (**F**,**G**) Transwell assay results showing the decrease in the migration in A549 cells upon the knockdown of BUB1B. (**H**,**I**) Western blot results showing the decrease in the EMT progression in A549 cells upon the knockdown of BUB1B. All experiments were repeated three times with three replicates for each repeat. * *p* < 0.05, ** *p* < 0.01, and *** *p* < 0.001.

Finally, to investigate the biological function of BUB1B in lung cancer progression, we subsequently performed loss-of-function experiments and silenced the expression of BUB1B in the human LUAD cell line A549. Western blot and RT-qPCR confirmed the efficiency of siRNA knockdown of BUB1B (Figure 7A,B). CCK8 assay for cell viability showed that the knockdown of BUB1B decreased the viability of A549 cells in vitro (Figure 7C). Wound healing (Figure 7D,E) and transwell migration (Figure 7F,G) assays demonstrated diminished migration in BUB1B knockdown cells. Moreover, results from the Western blot assay also showed that the knockdown of BUB1B significantly inhibits the EMT process in LUAD cells. It showed that BUB1B knockdown increased the expression of E-cadherin and decreased the expression of vimentin and N-cadherin (Figure 7H,I).

## 3. Discussion

BUB1B plays a central role in SAC signaling and stable attachment of kinetochores to the spindle microtubule. Recent studies have increasingly demonstrated a correlation between elevated BUB1B expression and the incidence and progression of various cancers. For instance, BUB1B contributes to the progression of hepatocellular carcinoma by activating the mTORC1 signaling pathway [21]. Additionally, BUB1B overexpression has been linked to increased tumorigenicity and resistance to radiotherapy in glioblastoma [26]. Moreover, BUB1B may contribute to drug resistance to bortezomib and adriamycin in multiple myeloma [20]. In this study, bioinformatics methods were used to elucidate the expression pattern, prognostic significance and potential function of BUB1B in various cancer types.

We found that BUB1B was significantly upregulated and was correlated with the clinical stage in several cancers, and the ROC curve analysis shows that BUB1B may function as a diagnostic biomarker. The prognostic analysis shows that increased BUB1B was related to poorer OS in ACC, KIRC, KIRP, LGG, LIHC, LUAD, MESO, PAAD, and SARC. BUB1B may function as a potential biomarker and therapy target of LUAD. Among them, we found out that BUB1B was significantly upregulated and correlated with the stage of LUAD. Thus, we chose LUAD to verify our bioinformatics result. In terms of the mechanism, the GO and KEGG analyses suggest that BUB1B was correlated with cell cycle and DNA replication, and the single-cell sequence data also suggest that BUB1B was positively associated with proliferation, DNA damage, DNA repair, cell cycle, and invasion. Meanwhile, our in vitro experiments suggest that the knockdown of BUB1B may significantly suppress the proliferation abilities of A549 cells and diminish migration. Furthermore, vimentin and N-cadherin were significantly downregulated and E-cadherin was significantly upregulated after the knockdown of BUB1B.

We analyzed BUB1B mutations in 32 cancer types using TCGA data. Uterine carcinosarcoma had the highest mutation rate, at 7.02%, followed by uterine corpus endometrial carcinoma (6.24%) and others. TMB has been identified as a potential biomarker for various cancers, as assessed by the total number of somatically encoded mutations [27,28]. Prior research has demonstrated that tumors exhibiting high tumor mutational burden (TMB) are responsive to immunotherapy and are linked to enhanced survival outcomes [29,30]. We found a positive correlation between BUB1B expression and TMB in several cancers, including ACC, KICH, CHOL, PAAD, SARC, LAML, SKCM, BLCA, COAD, and KIRC, but a negative correlation in ESCA and THYM. Microsatellite instability (MSI), caused by DNA mismatch repair deficiencies, is linked to higher cancer risk and is a key biomarker for immune checkpoint therapy [31,32]. Our study found a positive correlation between BUB1B expression and MSI in seven cancer types (STAD, LUSC, COAD, UCEC, SARC, LIHC, ACC) and a negative correlation in DLBC. Currently, BUB1B mutations are underreported in these cancers. Our research provides new insights into their mutational landscape.

Tumorigenesis is intricately linked to the tumor microenvironment (TME), and recent advancements have identified strategies targeting the TME as a promising avenue for cancer therapy. The immune cell composition within the TME, such as myeloid-derived suppressor cells (MDSCs), natural killer T cells (NKT), CD4+ T cells, and CD8+ T cells, plays a significant role in modulating the efficacy of immunotherapy [32,33,34,35,36,37]. Our findings indicated that the expression of BUB1B was positively correlated with the infiltration levels of MDSCs across various tumor types, with the most pronounced associations observed in ACC, LIHC, KICH, and LUAD. Furthermore, the findings indicate a negative correlation between the infiltration levels of T-cell NK and BUB1B expression across the majority of tumors. The four tumors with the highest correlation were UVM, UCEC, PRAD, and BLCA. In LUAD, BUB1B expression demonstrated a significant negative correlation with four other types of immune cell infiltration. As for LUAD, the obtained results show that there was a negative association between BUB1B and B cell, macrophage cell, CD4+ T cell, and CD8+ T cell infiltration. Furthermore, the single-cell sequence data also suggest that BUB1B was negatively correlated with the inflammation level of LUAD. Thus, BUB1B is related to the infiltration level of immune cells and may function as a potential immune therapy related biomarker in LUAD.

Recent research has established a link between myeloid-derived suppressor cells, NKT cells, and patient survival in lung cancer, as well as their response to immunotherapy [38,39]. Furthermore, macrophages, specifically tumor-associated macrophages (TAMs), are recognized as key constituents of the tumor microenvironment (TME). A transition from the pro-tumorigenic M2 phenotype to the tumoricidal M1 phenotype may present a valuable opportunity to enhance the efficacy of current cancer therapies.

Subsequently, we investigated the correlation between BUB1B expression and immunogenetics. MHC molecules play a critical role in the mechanisms underlying tumor immune evasion, as tumor cells can modulate MHC expression to facilitate immune escape, thereby promoting tumor progression and development. Numerous studies have demonstrated that a reduction in the expression of major histocompatibility complex (MHC) molecules enables tumor cells to evade detection and destruction by immune cells, thereby facilitating tumor proliferation and metastasis in cancers such as prostate cancer and melanoma. Consequently, investigating the role of MHC in tumorigenesis and tumor progression holds substantial significance. In Figure S3, our results showed that BUB1B had a negative correlation with MHC. So, BUB1B may reduce the recognition of immune cells by reducing the expression of immunogenetic molecules, thereby promoting tumor progression and deteriorating prognosis, which is not conducive to survival.

While bioinformatics analysis offer valuable insights into the role of BUB1B in malignancies, we substantiated its tumor promoting function in lung cancer through molecular biology techniques. Nonetheless, additional in vitro and in vivo experiments are necessary to corroborate our findings. Despite our efforts to analyze and integrate data from multiple databases, our study faced certain limitations. Notably, the TCGA database predominantly comprises data from Caucasian patients, resulting in a relative scarcity of information from patients of other ethnicities. In the loss-of-function experiment, our findings demonstrate that the knockdown of BUB1B resulted in diminished viability and inhibition of migration and the EMT process in LUAD cells. In conclusion, the findings of the present study indicate that BUB1B expression significantly influences the progression of lung cancer. Consequently, BUB1B holds potential as a biomarker for predicting patient prognosis and the efficacy of anticancer therapies in lung cancer.

## 4. Materials and Methods

### 4.1. Analysis of the Expression Level of BUB1B in the Pan-Cancer Datasets

The Human Protein Atlas (HPA) was utilized to investigate the expression and subcellular localization of the BUB1B protein across various organs. Immunofluorescence staining images of BUB1B in three human cancer cell lines (A-431, U-251MG, and U2OS) were also retrieved from the Human Protein Atlas (HPA; www.proteinatlas.org, accessed on 16 September 2024) [40] online platform. The expression levels of the BUB1B gene in a range of cancer tissues were acquired using the “Gene_DE” module available in TIMER 2.0 (http://timer.cistrome.org/, accessed on 16 September 2024) [41]. The association between BUB1B and cancer stage was analyzed with the “Stage plots” module of GEPIA2 (http://gepia2.cancer-pku.cn/#index, accessed on 16 September 2024) [38]. The 33 types of cancer, along with their full names and abbreviations, are detailed in Table S1.

### 4.2. Identification of the Correlation Between BUB1B Expression Levels and Diagnosis and Prognosis Analysis in Human Cancers

The potential value of BUB1B in cancer diagnosis was detected with the ROC curve with the data from the TCGA database. AUC > 0.85 was thought of as a high diagnostic value. The relationship between BUB1B expression and the prognosis of patients across various cancer types was analyzed using overall survival (OS) metrics. The GEPIA2 platform (http://gepia2.cancer-pku.cn/#index, accessed on 24 September 2024) [42] was utilized to examine cancer prognosis.

### 4.3. Association Between BUB1B Expression and Mutation

The mutational characteristics of BUB1B across various cancers were examined using the cBioPortal platform (http://www.cbioportal.org/, accessed on 26 September 2024) [43]. The “TCGA Pan Cancer Atlas Studies” cohort was selected for this analysis. Subsequently, “BUB1B” was input into the “Query” module. Information regarding the alteration sites, types, and frequencies of BUB1B mutations was accessible within the “cancer type summary” and “mutation” modules. Variation data was obtained from the Genomic Data Commons (GDC) portal (https://portal.gdc.cancer.gov/, accessed on 26 September 2024) [44] and subsequently processed utilizing the MuTect2 (version 4.1.6)and GISTIC software (version 2.0) tools. Statistical analysis was conducted using R software, version v4.0.3. Tumor mutational burden (TMB) metrics were sourced from the study by Vesteinn Thorsson et al. [45], while microsatellite instability (MSI) values were derived from the research conducted by Russell Bonneville et al. [46].

### 4.4. Analysis of the Correlation Between BUB1B and Immune Infiltration

The “GENE” module within the TIMER database (http://timer.cistrome.org/, accessed on 14 October 2024) [41] was employed to assess the infiltration levels of immune cells across 32 distinct cancer types. Additionally, the TISIDB platform (http://cis.hku.hk/TISIDB/, accessed on 14 October 2024) [47] was utilized to examine the correlation between BUB1B and immunogenetics.

### 4.5. Gene Co-Expression Analysis

A gene co-expression analysis of BUB1B was conducted utilizing the LinkedOmics database (https://www.linkedomics.org/login.php, accessed on 18 October 2024) [48]. For this analysis, the “HiSeqRNA” platform and the “TCGA_LUAD” cohort were selected. The Pearson correlation test was employed to assess the relationship between BUB1B and its co-expressed genes.

### 4.6. Single-Cell Sequence Analysis

The correlation between BUB1B and 14 cancer functional states were analyzed using single-cell sequence data from the “correlation plot” module of the CancerSEA website (http://biocc.hrbmu.edu.cn/CancerSEA/, accessed on 21 October 2024) [49].

### 4.7. Cell Culture and siRNA Transfection

The human lung cancer cell line A549 was cultured in RPMI 1640 medium (Gibco, Shanghai, China) supplemented with 10% fetal bovine serum (FBS), 1% L-glutamine, and 1% penicillin–streptomycin (Gibco, CA, USA). The cell cultures were maintained at 37 °C in a CO_2_ incubator (Thermo Fisher Scientific, Waltham, MA, USA). Transfection of A549 cells with si-NC and si-BUB1B small interfering RNA (siRNA) were performed using the Lipo8000 transfection reagent (Beyotime, Shanghai, China), following the manufacturer’s instructions. The siRNA were as follows: si-BUB1B: 5′-CCAGUUCUGUUUGUCAAGUAATT-3′, si-NC: 5′-TTCTCCGAACGTGTCACGUTT-3′.

### 4.8. RNA Isolation and qPCR

Following a 48-h transfection period, the mRNA expression levels of BUB1B were quantified using a quantitative PCR (qPCR) assay. In summary, total RNA was isolated from the cells utilizing the Total RNA Extraction Kit (Kangwei, Wuhan, China). Subsequently, 2 µg of RNA were reverse transcribed into complementary DNA (cDNA), and the mRNA levels were assessed using the UltraSYBR Master Mix (CWBIO, Guangzhou, China). The qPCR primers specific for BUB1B were as follows: F-GTCTCGGCAAACTCTGTTGG and R-TCCTCTGTTCTGGCTTGGAG; and GAPDH: F-GACAGTCAGCCGCATCTTCT and R-TTAAAAGCAGCCCTGGTGAC.

### 4.9. Western Blotting

Proteins were extracted from cells utilizing RIPA lysis buffer (Beyotime, Shanghai, China). The resulting lysates were subjected to SDS-PAGE for separation. Following electrophoresis, the proteins were transferred onto a PVDF membrane. The membrane was subsequently blocked with 5% non-fat milk and incubated sequentially with primary and secondary antibodies. Protein detection was facilitated using enhanced ECL buffer (Beyotime, Shanghai, China), and the signals were captured with a ChemiDocXRS + Imaging System (Bio-Rad, Hercules, CA, USA). Quantitative analysis of the protein bands was conducted using Image Lab software Version 6.1 (Bio-Rad, CA, USA). The information for antibodies was as follows: BUB1B (Boster, Wuhan, China), vimentin (CST, Danvers, MA, USA), E-cadherin (CST, MA, USA), N-cadherin (CST, MA, USA), and GAPDH (CST, MA, USA).

### 4.10. Cell Proliferation

We inoculated each well of 96-well plates with 3 × 10^3^ cells, supplemented with 100 µL of culture medium. The cells were incubated under standard conditions, and a CCK-8 assay (Abbkine Scientific Co., Wuhan, China) was conducted at 24, 48, and 72 h post-initial attachment. Optical density (OD) values were subsequently measured at 450 nm using a microplate reader.

### 4.11. Wound-Healing Assay

We further assessed the impact of BUB1B on cellular migration through the wound-healing assay. In summary, a wound was induced in a 6-well plate by scratching the surface with a 200 µL pipette tip. The wounded regions were imaged using a light microscope (Nikon, Tokyo, Japan) immediately after wound creation (0 h) and again at 24 h post-wounding. The percentage of wound closure was determined using the formula: [1 − (empty area at 24 h/empty area at 0 h)] × 100%.

### 4.12. Cell Migration Assay

Cell migration was evaluated utilizing transwell chambers with 8-µm pores (Corning Inc., Corning, NY, USA). Cell suspensions, consisting of 1 × 10^4^ cells in serum-free medium, were introduced into the upper chamber. Following a 24 h incubation period at 37 °C, the cells that had migrated were fixed with 4% paraformaldehyde for 20 min and subsequently stained with 0.5% crystal violet for an additional 20 min at room temperature. The stained cells were then quantified using a light microscope (Nikon, Tokyo, Japan).

### 4.13. Statistical Analysis

The data were analyzed and visualized utilizing GraphPad Prism 7, with results expressed as the mean ± standard deviation (SD) from three independent replicates. Comparative analysis between two groups was conducted using Student’s *t*-test, with a *p*-value of less than 0.05 considered indicative of statistical significance.

## 5. Conclusions

In conclusion, our comprehensive pan-cancer analysis demonstrated that BUB1B is aberrantly expressed across various cancer types, with its expression levels significantly associated with clinicopathological characteristics and patient prognosis, particularly in lung adenocarcinoma. Specifically, BUB1B was found to be upregulated in LUAD, correlating with clinical diagnosis and prognosis and tumor immunity. These findings suggest that BUB1B may serve as a valuable prognostic and immunological biomarker for LUAD.

## Data Availability

The data that support the findings of this study are available from the corresponding author upon reasonable request.

## Supplementary Materials

The following supporting information can be downloaded at: https://www.mdpi.com/article/10.3390/ijms26052061/s1.
