# Supplementary material for: A Comprehensive Analysis Revealing BUB1B as a Potential Prognostic and Immunological Biomarker in Lung Adenocarcinoma
Source: Int J Mol Sci. 2025 Feb 26;26(5):2061. doi: 10.3390/ijms26052061 (PMC11899737; doi:10.3390/ijms26052061)
Supplement: Supplementary file 1 [file ijms-26-02061-s001.zip › ijms-3411333-supplementary.pdf]

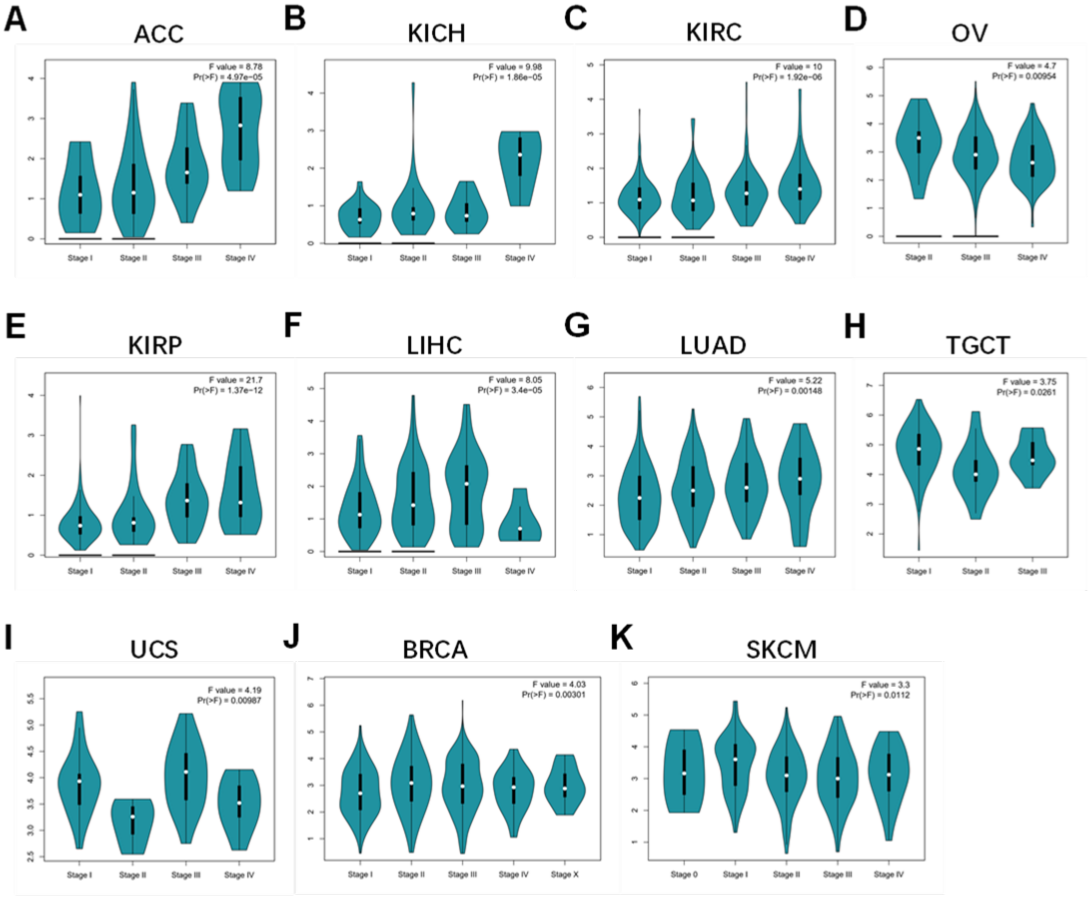

Figure S1. The correlation between BUB1B expression and the pathological stages. GEPIA2 database analyses of the association between BUB1B expression and the pathological stages in (A)ACC, (B)KICH, (C)KIRC, (D)OV, (E)KIRP, (F)LIHC, (G)LUAD, (H)TGCT, (I)UCS, (J)BRCA and (K)SKCM.

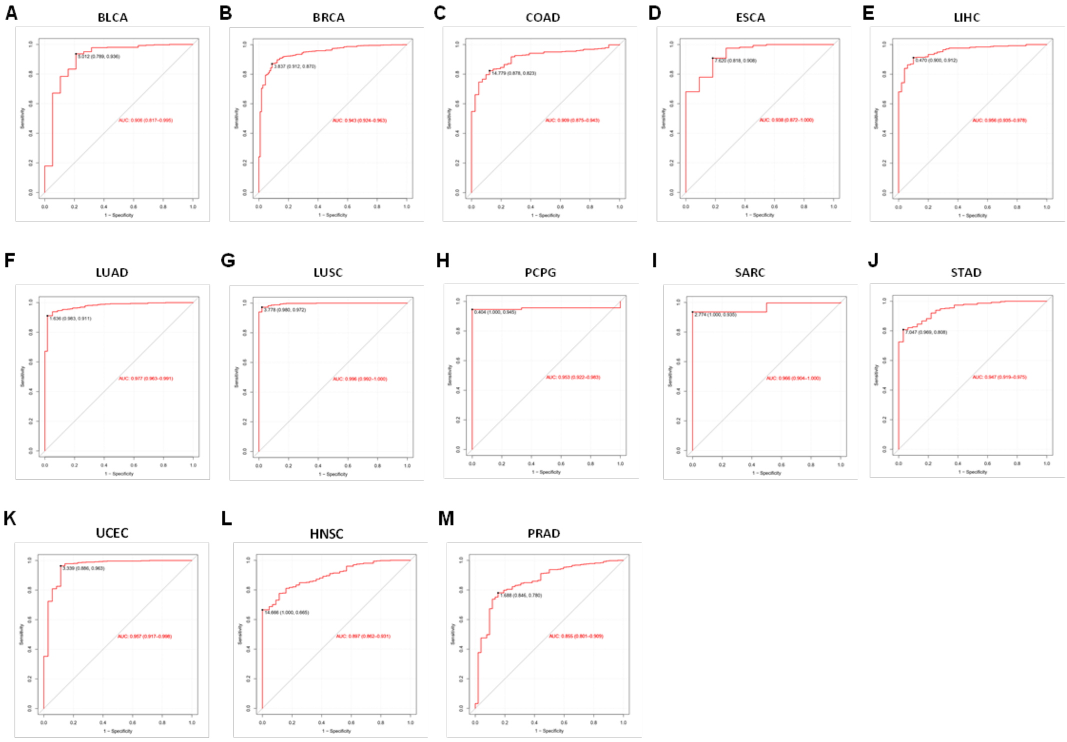

Figure S2. The diagnostic value of BUB1B in pan-cancer. (A–M). The ROC curves of BUB1B in BLCA, BRCA, COAD, ESCA, LIHC, LUAD, LUSC, PCPG, SARC, STAD, UCEC, HNSC, PRAD.

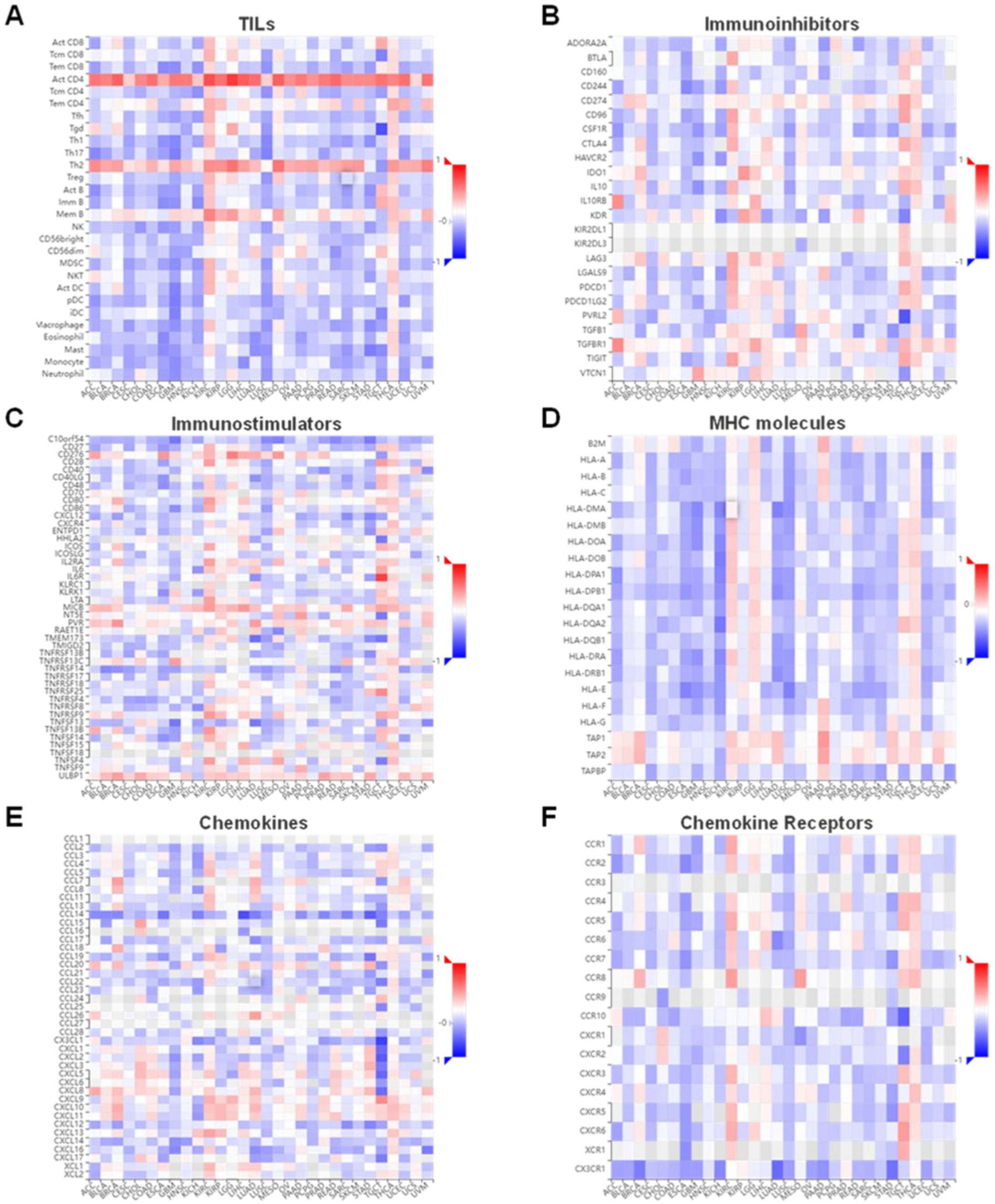

Figure S3. Heatmap analysis of the correlation between BUB1B expression and (A) TILs, (B) immunoinhibitors, (C) immunostimulators, (D) MHC molecules, (E) chemokines and (F) chemokines receptors in tumours.

Table S1. Abbreviations

Abbreviations	Full names
ACC	Adrenocortical carcinoma
BLCA	Bladder Urothelial Carcinoma
BRCA	Breast invasive carcinoma
CESC	Cervical squamous cell carcinoma and endocervical adenocarcinoma
CHOL	Cholangiocarcinoma
COAD	Colon adenocarcinoma
COADREAD	Colon adenocarcinoma/Rectum adenocarcinoma Esophageal carcinoma
DLBC	Lymphoid Neoplasm Diffuse Large B-cell Lymphoma
ESCA	Esophageal carcinoma
FPPP	FFPE Pilot Phase II
GBM	Glioblastoma multiforme
GBMLGG	Glioma
HNSC	Head and Neck squamous cell carcinoma
KICH	Kidney Chromophobe
KIPAN	Pan-kidney cohort (KICH+KIRC+KIRP)
KIRC	Kidney renal clear cell carcinoma
KIRP	Kidney renal papillary cell carcinoma
LAML	Acute Myeloid Leukemia
LGG	Brain Lower Grade Glioma
LIHC	Liver hepatocellular carcinoma
LUAD	Lung adenocarcinoma
LUSC	Lung squamous cell carcinoma
MESO	Mesothelioma
OV	Ovarian serous cystadenocarcinoma
PAAD	Pancreatic adenocarcinoma
PCPG	Pheochromocytoma and Paraganglioma
PRAD	Prostate adenocarcinoma
READ	Rectum adenocarcinoma
SARC	Sarcoma
SKCM	Skin Cutaneous Melanoma
STAD	Stomach adenocarcinoma
STES	Stomach and Esophageal carcinoma
TGCT	Testicular Germ Cell Tumors
THCA	Thyroid carcinoma
THYM	Thymoma
UCEC	Uterine Corpus Endometrial Carcinoma
UCS	Uterine Carcinosarcoma
UVM	Uveal Melanoma
BUB1B	BUB1 mitotic checkpoint serine/threonine kinase B
TCGA	The Cancer Genome Atlas
OS	Overall Survival
HPA	The Human Protein Atlas

GDC	the Genomic Data Commons
TME	Tumor microenvironment
ICIs	Immune checkpoint inhibitors
lncRNAs	Long non-coding RNAs
ROC	Receiver Operating Characteristic Curve
AUC	Area Under Curve
TMB	Tumor Mutational Burden
MSI	Microsatellite Instability
MDSCs	Myeloid-derived suppressor cells
NKT	Natural killer T cell
MHC	Major Histocompatibility Complex
EMT	Epithelial-mesenchymal transition
OD	Optical density
